# A Biochemical Analysis of the Interaction of *Porphyromonas gingivalis* HU PG0121 Protein with DNA

**DOI:** 10.1371/journal.pone.0093266

**Published:** 2014-03-28

**Authors:** Natalia O. Tjokro, Christopher J. Rocco, Richa Priyadarshini, Mary E. Davey, Steven D. Goodman

**Affiliations:** 1 Department of Molecular & Computational Biology, University of Southern California, Los Angeles, California, United States of America; 2 Department of Molecular Genetics, The Forsyth Institute, Boston, Massachusetts, United States of America; 3 Center for Microbial Pathogenesis, The Research Institute at Nationwide Children's Hospital, Columbus, Ohio, United States of America; University of Oklahoma Health Sciences Center, United States of America

## Abstract

K-antigen capsule, a key virulence determinant of the oral pathogen *Porphyromonas gingivalis*, is synthesized by proteins encoded in a series of genes transcribed as a large polycistronic message. Previously, we identified a 77-base pair inverted repeat region with the potential to form a large stem-loop structure at the 5′ end of this locus. *PG0121*, one of two genes flanking the capsule operon, was found to be co-transcribed with the operon and to share high similarity to the DNA binding protein HU from *Escherichia coli.* A *null* mutation in *PG0121* results in down-regulation of transcription of the capsule synthesis genes and production of capsule. Furthermore, we have also shown that PG0121 gene can complement multiple deficiencies in a strain of *E. coli* that is deficient for both the alpha and beta subunits of HU. Here, we examined the biochemical properties of the interaction of PG0121 to DNA with the emphasis on the kinds of nucleic acid architectures that may be encountered at the 77-bp inverted repeat. We have concluded that although some DNA binding characteristics are shared with *E. coli* HU, HU PG0121 also shows some distinct characteristics that set it apart from other HU-like proteins tested to date. We discuss our results in the context of how PG0121 may affect the regulation of the K-antigen capsule expression.

## Introduction

Prokaryotes must compact and organize their entire genomic DNA into a limited cellular space. To perform these functions, prokaryotes synthesize various nucleoid-associated proteins (NAPs), including the DNABII family of proteins, whose members consist of the dimeric histone-like HU proteins and their sequence-specific homolog Integration Host Factor (IHF). These NAPs not only associate with the genomic DNA to form bacterial chromatin [1] but also regulate various DNA metabolic processes, such as replication, recombination, repair, and transcription [2]. HU alleles are ubiquitous, with approximately 98% of sequenced prokaryote genomes encoding at least one allele [3].

Perhaps the most extensively studied family members are from *E. coli*, which possesses two HU alleles, the *hupA* and *hupB* genes, which encode the alpha and beta subunits [4]. These two subunits form both homodimers and heterodimers, with strong phenotypes only associated with deletion of both alleles [5]–[9]. When bound to DNA, HU acts as an architectural protein that coils double-stranded DNA into a nucleosome-like structure [10]. In fact, HU has the ability to introduce negative supercoiling into relaxed circular DNA molecules in the presence of topoisomerase I [11]–[13]. In addition, *E. coli* HU binds non-specifically to approximately 9 base pair sites in a double-stranded DNA molecule with an equilibrium dissociation constant in the micromolar range [14]. This affinity increases significantly for distorted DNA structures such as replication forks, three- or four-way junctions, nicks, overhangs, and gaps [15], [16] due to the lower energy required to distort the final DNA architecture [15]–[20]. This distortion is particularly evident in the presence of T4 DNA ligase, as *E. coli* HU has the ability to mediate very tight DNA curvature, causing cyclization of DNA fragments that are shorter than their persistence length (P) [21]. Finally, HU is also capable of binding RNA and associating with ribosomes [22]–[24].

HU's capacity to regulate specific *E. coli* genes is well known [25]. A previous study demonstrated that *E. coli* HU protein is involved in the negative regulation of the *hupA* and *hupB* genes [25]. The promoter region of the *E. coli hupA* gene overlaps a large inverted repeat region that has the potential to form a cruciform DNA structure [26]. Inverted repeat sequences embedded in negatively supercoiled DNA have been shown to be preferentially extruded to form a cruciform DNA structure because of the resulting reduction in the free energy of the negative supercoiling [27]. HU protein may facilitate the formation of this cruciform structure at least in part by increasing the negative superhelical density of the promoter region [26]. The expression of the *hupA* gene is negatively regulated by the potential steric hindrance imposed by this cruciform structure on the functional promoter domains of the gene. This structure may prevent RNA polymerases from accessing the transcription initiation signals.

**Figure 1:**
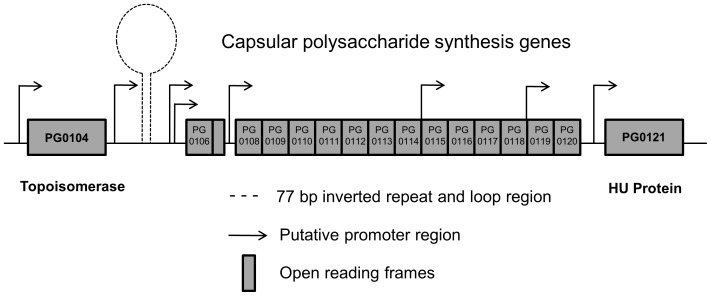
Schematic of the inverted repeat region upstream of the K-antigen capsule operon. The K-antigen capsule is encoded by a series of genes in an operon (*PG0106* – *PG0120*). Two genes flanking this operon, *PG0104* and *PG0121*, are predicted to encode DNA binding proteins due to their high similarity to the known DNA binding proteins. They are oriented in the same direction as the capsule operon, and they are being co-transcribed to yield multiple transcripts, including one large polycistronic message encoding the entire region from *PG0104* – *PG0121*. Adapted from [28].

Interestingly, a similar inverted repeat region with the potential to form a large stem loop structure was also found upstream of the K-antigen capsule operon in *P. gingivalis*, the etiological agent of severe forms of periodontitis [28]. Our recently published study reported the presence of a possible HU homolog in *P. gingivalis* that is encoded by gene PG0121. The PG0121 gene is in the same orientation as and co-transcribed with the K-antigen capsule operon [28], a key virulence factor for *P. gingivalis* [29]–[34]. The 77-bp inverted repeat region was found upstream of the start site of PG0106, which is the first gene in the K-antigen capsule operon (Figure 1), and it is predicted to form a large stem loop structure with a predicted free energy of dissociation of −126 kcal/mol [28]. PG0121 can also complement some of the functions of HU in an *E. coli* strain that is deficient for both subunits of HU [35]. Additionally, *E. coli* HU can bind to secondary structures that may form via this stem loop structure [36].

**Figure 2:**
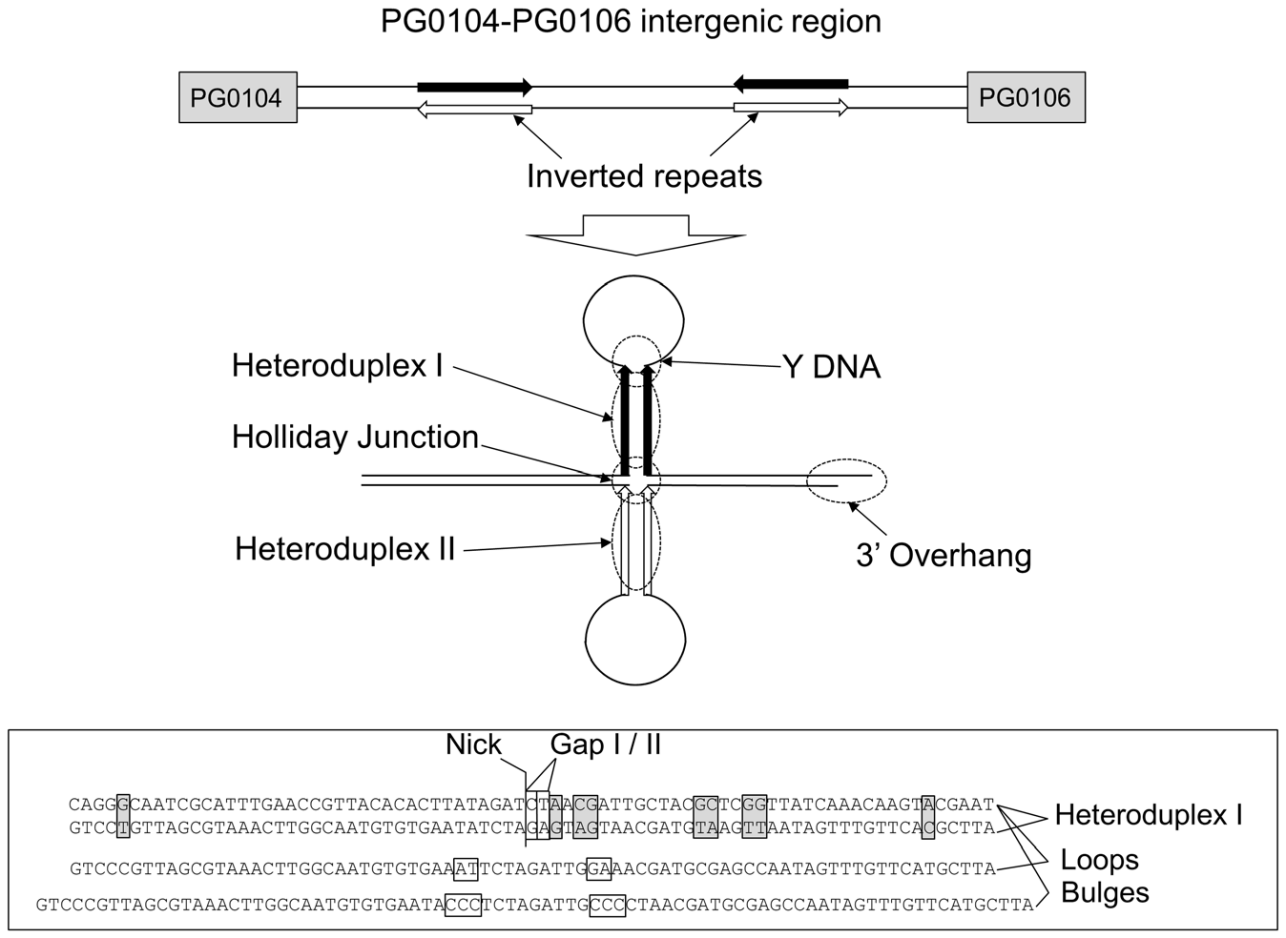
Schematic of cruciform and potential structures for HU PG0121 binding. The top shows the intergenic region between *PG0104* and *PG0106* with inverted repeats leading to the potential cruciform structure shown in the center. Location of potential structures for HU PG0121 binding are circled and labelled. Insert box shows the DNA sequence of the 77 bp Heteroduplex I structure with mismatches highlighted. Locations of the alterations in the Nick, Gap I, Gap II, Bulges, and Loop structures are shown as well.

Although it has been established that there is a link between the production of capsule and the presence of gene PG0121 [28], the molecular mechanisms underlying this regulation have not been determined. The location of the large 77-bp inverted repeat upstream of the capsule operon suggests that this region may be involved in regulation. Previously, we showed that a His-affinity tagged HU PG0121 protein binds to DNA containing this inverted repeat [28]; yet it remains unknown whether this binding is sequence-specific and/or specific for particular DNA secondary structures [28]. Here, we performed a more thorough analysis of PG0121 DNA binding characteristics, particularly in the absence of an affinity tag, which could obscure full function. To perform these experiments, we did binding analysis of tagless HU PG0121 to DNA molecules possessing both sequences and potential structures that are associated with the region upstream of the capsule operon, including the 77-bp inverted repeat. A schematic of the potential structures examined in this study is shown in Figure 2. We also examined the known DNA binding properties of *E. coli* HU to characterize other possible functions that could affect gene regulation, including DNA bending, secondary structure preference and restraint of DNA supercoils. These characteristics were then compared to other HU homologs to assess the similarity of HU PG0121 to other HU-like proteins.

## Materials and Methods

### Cloning, overexpression and purification of tagless HU PG0121 protein

Tagless HU PG0121 was cloned using chromosomal DNA from *P. gingivalis* strain W83 and the Intein Mediated Purification with an Affinity Chitin-binding Tag (IMPACT) kit (New England Biolabs, Ipswich, MA, USA) following the manufacturer's protocol. Briefly, the coding region for PG0121 was PCR-amplified from *P. gingivalis* strain W83 chromosomal DNA using primers oSG695 and oSG696 (Table 1), which introduced NdeI and SapI restriction sites to the PCR product. The PCR products were amplified using Herculase II Fusion DNA polymerase (Agilent Technologies, Santa Clara, CA, USA) prior to digestion with the restriction enzymes NdeI and SapI (New England Biolabs) and subsequent purification using the QIAquick Gel Extraction Kit (Qiagen). The HU PG0121 amplicon was then ligated into pTXB1 (New England Biolabs) at the NdeI and SapI sites, transformed into *E. coli* ER2566 cells and selected for ampicillin resistance. The plasmids were then confirmed by sequencing.

**Table 1:** Sequences of oligonucleotides for DNA substrates used in this study.

oSG	Sequence of Oligonucleotides (5′ – 3′)
309	TTTTTTATAATGCCAACTTAGTATAAAAAAGCTGAACGAGAAACGTAAAA
310	TTCCCGTTTCGCTCAAGTTAGTATAAAAAAGCAGGCTTCAACGGATTCAT
311	ATGAATCCGTTGAAGCCTGCTTTTTTATACTAAGTTGGCATTATAAAAAA
606	TTTTACGTTTCTCGTTCAGCTTTTTTATACTAACTTGAGCGAAACGGGAA
607	TTCCCGTTTCGCTCAAGGTTGGCATTATAAAAAA
608	TTCCCGTTTCGCTCAAGTTAGTATAAAAAAGCTGAACGAGAAACGTAAAA
561	CAGGGCAATCGCATTTGAACCGTTACACACTTATAGATCTAACGATTGCTACGCTCGGTTATCAAACAAGTACGAAT
562	ATTCGCACTTGTTTGATAATTGAATGTAGCAATCATGAGATCTATAAGTGTGTAACGGTTCAAATGCGATTGTCCTG
565	CAGGGCAATCGCATTTGAACCGTTACACACTTATAGATCTAACGATTGCTACGCTCGGTTATCAAACAAGTACGAATTAATGGGACCTTTGCACAAT
592	ATTCGTACTTGTTTGATAACCGAGCGTAGCAATCGTTAGATCTATAAGTGTGTAACGGTTCAAATGCGATTGCCCTG
640	TAACAATCTCTCAGATTAGGATTCGTACTTGTTTGATAACCGAGCGTAGCAATCGTTAGATCTATAAGTGTGTAACGGTTCAAATGCGATTGCCCTG
641	ATTGTGCAAAGGTCCCATTAATTCGTACTTGTTTGATAACCGAGCGTAGCAATCGTTAGATCTATAAGTGTGTAACGGTTCAAATGCGATTGCCCTG
642	CAGGGCAATCGCATTTGAACCGTTACACACTTATAGATCTAACGATTGCTACGCTCGGTTATCAAACAAGTACGAATCCTAATCTGAGAGATTGTTA
695	GGTGGTCATATGAACAAGACAG
696	CTTTGGAACTTAAGTGCGGAAGAGCAACCACC
795	ATTCGTACTTGTTTGATAACCGAGCGTAGCAAAGGTTAGATCTTAAAGTGTGTAACGGTTCAAATGCGATTGCCCTG
796	ATCTATAAGTGTGTAACGGTTCAAATGCGATTGCCCTG
797	ATTCGTACTTGTTTGATAACCGAGCGTAGCAATCGTTAG
798	ATTCGTACTTGTTTGATAACCGAGCGTAGCAATCGTTA
799	ATTCGTACTTGTTTGATAACCGAGCGTAGCAATCGTT
800	GTACTTGTTTGATAACCGAGCGTAGCAATCGTTAGATCTATAAGTGTGTAACGGTTCAAATGCGATTGCCCTG
801	ATTCGTACTTGTTTGATAACCGAGCGTAGCAATCCCCGTTAGATCTCCCATAAGTGTGTAACGGTTCAAATGCGATTGCCCTG
836	CAGGACAATCGCATTTGAACCGTTACACACTTATAGATCTCATGATTGCTACATTCAATTATCAAACAAGTGCGAAT
873	CAGGTCAATCGCATTTGAACCGTTACACACTTATAGATCTGAAGATTGCTACTATCTTTTATCAAACAAGTCCGAAT
874	ATTCGTACTTGTTTGATAACCGAGTGTAGCAATCATTAGATCTATAAGTGTGTAACGGTTCAAATGCGATTGCCCTG
875	ATTCGTACTTGTTTGATAATCGAGCGTAGCAATCGTGAGATCTATAAGTGTGTAACGGTTCAAATGCGATTGCCCTG
876	CAGGACAATCGCATTTGAACCGTTACACACTTATAGATCTCACGATTGCTACGTTCAATTATCAAACAAGTGCGAAT
877	CAGGACAATCGCATTTGAACCGTTACACACTTATAGATCTAATGATTGCTACATTCAGTTATCAAACAAGTGCGAAT
878	CCATGGATCCGAGCTCGAG
879	GATGATGGATCCATGATGGTCG
881	ATTCGCACTTGTTTGATAATTGAATGTAGCAATCATGAGATCTATAAGTGTGTAACGGTTCAAATGCGATTGTCCTG
882	ATTCGTACTTGTTTGATAACCGAGCGTAGCAATCGTTAGATCTATAAGTGTGTAACGGTTGTAATGCGATTCGCCTG
883	ATTCGTACTTGTTTGATAACCGAGCGTAGCAATCGTTAGATCTATAAGTCAGTAACGGTTGTAATGCGATTGCCCTG
884	ATTCGTACTTGTTTGTAAACCGAGCGATGCAATCGTTAGATCTATAAGTGTGTAACGGTTCAAATGCGATTGCCCTG
885	ATTCCAACTTGTTTGTAAACCGAGCGTAGCAATCGTTAGATCTATAAGTGTGTAACGGTTCAAATGCGATTGCCCTG
886	GATCTAACGATTGCTACGCTCGGTT
887	AACCGAGCGTAGCAATCGTTAGATC
77-mer Control	TTTCCCATTATAATAATAAAAAAACAATGATGTTATAGAACTGTAATAAGTTTGTTGCTTAGAATTTATAGTTTGAT

Tagless HU PG0121 protein was first purified by chitin affinity chromatography from a 500-mL liquid culture of *E. coli* ER2566 carrying pTXB1 with the HU PG0121 gene. When the OD_600 nm_ reached approximately 0.3–0.5, the culture was induced with 1 mM isopropyl β-D-1-thiogalactopyranoside (IPTG) for 3 hours. The cells were harvested at 5,000× *g* for 15 minutes and resuspended in 10 mL of cold column buffer (20 mM 2-amino-2-(hydroxymethyl)propane-1,3-diol hydrochloride (Tris-HCl) pH 8.5, 0.5 mM NaCl). Sarkosyl was added to a final concentration of 0.5%, and the mixture was sonicated on ice for 6×30-second cycles with 1-minute rest intervals. The supernatant was diluted in column buffer to obtain a final concentration of 0.05% Sarkosyl, loaded onto a chitin column at 4°C, and washed with at least 20 column volumes of column buffer. The column was then washed with 3 column volumes of cleavage buffer (20 mM Tris-HCl pH 8.5, 0.5 mM NaCl, 50 mM DTT) and incubated at 4°C for 16–40 hours. Column buffer was added, and samples were eluted in 1-mL fractions.

Secondary purification of the HU protein was then performed using P11 phosphocellulose cation exchange chromatography. The protein-containing fractions that were collected from the chitin column were combined and diluted in cold TG buffer (50 mM Tris-HCl pH 7.4, 10% glycerol). The mixture was then loaded onto a P11 phosphocellulose column that was prepared following manufacturer's protocol, washed with TG buffer containing 0.4 M KCl, eluted with TG buffer containing 1.2 M KCl, and dialyzed overnight against cold sterile water. The protein concentrations were determined using the Protein Assay reagent (Bio-Rad, Hercules, CA, USA) with bovine serum albumin as a standard, and the protein was purified to approximately 95% homogeneity.

### Crosslinking of the HU PG0121 protein

Crosslinking of the HU PG0121 protein was performed as previously described [37]. Briefly, increasing concentrations of the HU PG0121 protein were incubated with 0.1% glutaraldehyde in 10 mM sodium phosphate buffer (pH 7.0) for 30 minutes at room temperature in a total reaction volume of 10 µL. Equal volumes of 2× Laemmli sample buffer were added, and the samples were analysed using polyacrylamide gel electrophoresis and Coomassie blue staining.

### Labelling of DNA probes

**Figure 3:**
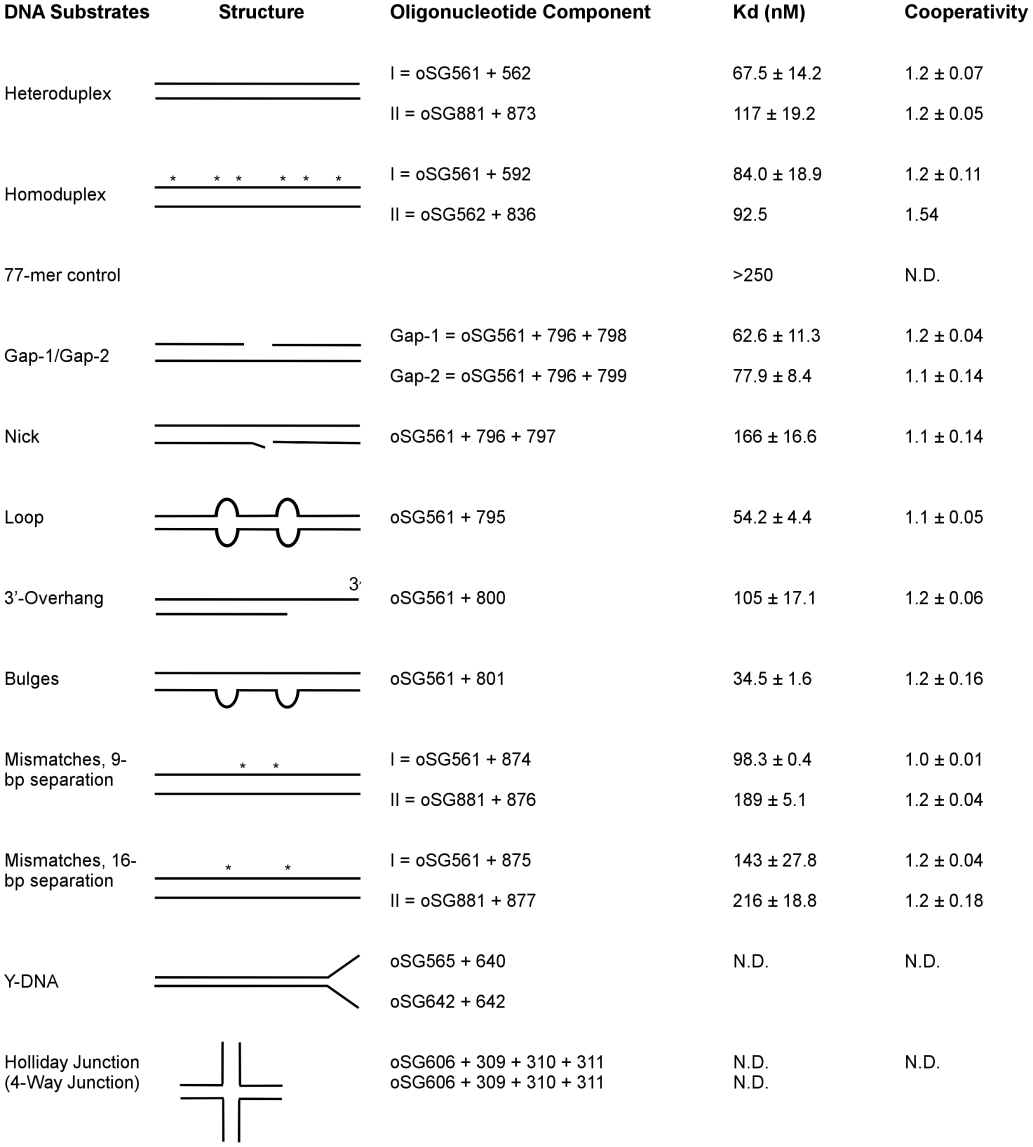
Structures, K_d_, and cooperativity values of DNA substrates used in the characterization HU PG0121 protein. Asterisks signify the mismatches in the sequence. The values were determined from at least two experiments with the exception of the homoduplex II and the 77-mer control DNA substrates, which were performed only once. The errors were determined by Standard Error of the Mean (SEM). N.D. – values were not determined.

The oligonucleotide sequences used in this study are listed in Table 1, and the DNA probes used in this study are listed in Figure 3. The labelling of the DNA probes was performed as previously described [38]. In brief, a 0.5 µM stock of the appropriate oligonucleotide was isotopically labelled at the 5′-end with 0.034 µM of [γ-^32^P] ATP (10 mCi/ml) (Perkin Elmer, Waltham, MA, USA) and 10 units of T4 polynucleotide kinase (New England Biolabs). The oligonucleotide was then purified on an Illustra MicroSpin G-50 column (GE Healthcare, Piscataway, NJ, USA) following the manufacturer's protocol. Each DNA substrate was annealed by heating 0.5 µM of the ^32^P-labeled oligonucleotide with a 0.4 µM solution of the appropriate unlabelled oligonucleotides from 10 uM stock solutions in 10 mM Tris-HCl (pH 8.0) containing 100 mM NaCl for 3 minutes at 100°C and allowing the reaction to cool to room temperature in 2.5–3 hours. The annealed DNA substrates were drop-dialyzed against 4 mL of 1× TE (10 mM Tris-HCl, 1 mM ethylenediaminetetraacetic acid (EDTA)) for 10 minutes using a 25 mm 0.025 µm nitrocellulose membrane disc (Millipore, Billerica, MA, USA), and the Holliday Junction DNA substrate was purified using the crush and soak method as previously described [39].

### Electrophoretic Mobility Shift Assay (EMSA)

EMSAs were performed as previously described [38]. Briefly, the appropriate protein was incubated with the labelled DNA substrates in reaction buffer (52.5 mM *N*-(2-hydroxyethyl)-piperazine-*N*′-2-ethanesulfonic acid (HEPES) pH 6.5, 50 µM EDTA, 9.5% glycerol, 100 mM KCl, and 50 µg/ml BSA) for 30 minutes at room temperature. The reaction mixtures were then separated using 6% non-denaturing polyacrylamide gel electrophoresis in 0.5× TBE (45 mM Tris, 45 mM boric acid, 1 mM EDTA) running buffer at 10 V/cm for 3 hours. The HJ DNA substrate was electrophoresed for 6 hours. The gels were dried and scanned using either the GE Healthcare Typhoon FLA-7000 (Pittsburgh, PA, USA) or the Bio-Rad PharosFX Molecular imaging system.

To calculate the dissociation constants and the binding cooperativity values, ImageQuant 5.0 software (Molecular Dynamics, Sunnyvale, CA, USA) was used to quantify the band intensities of the shifted and free DNA in each reaction. The dissociation constants were calculated using the GraphPad Prism 4.0 software (San Diego, CA, USA). The ratios of shifted/total DNA were plotted against the protein concentrations and fitted by non-linear regression using the one-site binding (hyperbola) equation, which describes the equilibrium binding of a ligand (i.e., HU PG0121) to a receptor (i.e., DNA substrate) as a function of increasing ligand concentration. In contrast, the dissociation constant for the independent 77-mer DNA control substrate was calculated based on the disappearance of the substrate. For cooperativity values, the log of the ratio of shifted/free DNA was plotted against the log of the total protein concentration. The resulting slope is the Hill coefficient.

### HU PG0121 and heat stability of HJ DNA substrates

EMSA was performed as previously described [38] with slight modifications. After an initial 30-minute incubation at room temperature, one reaction was subjected to a 10-minute treatment at 55°C, while the control group was left at room temperature. The reaction mixtures were then separated using 6% non-denaturing polyacrylamide gel electrophoresis in 0.5× TBE running buffer at 10 V/cm for 2 hours. The gels were dried and scanned as described in the previous section.

### Branch migration assay

A branch migration assay was performed as previously described [40], with modifications. Labelled *Y-DNA substrate (*oSG565 + oSG640), which is the homoduplex form of the 77-bp inverted repeat sequence with 20-nucleotide non-complementary single-stranded tails, was incubated with a 5 M excess of a second unlabelled Y-DNA substrate (oSG642 + 641), which is the same homoduplex form of the 77-bp inverted repeat sequence, but the 5′-end of the 20-nucleotide single-stranded tails complements the 3′-end of the single-stranded tails from the labelled Y-DNA substrate, and with the designated concentration of protein in 20 µL of reaction buffer containing 52.5 mM HEPES pH 6.5, 0.05 mM EDTA, 9.5% glycerol, 50 µg/ml BSA, and 100 mM MgCl_2_. The reaction mixture was then separated using non-denaturing 6% acrylamide gel electrophoresis. The tail region of the *Y-DNA was specifically designed to be complementary to the tail region of the Y-DNA substrate. Upon annealing, the two partial duplexes (*Y and Y) would be joined to form a four-stranded complex (HJ), and the HJ branches would migrate by random walk until 2 thermodynamically stable homoduplexes formed.

### Supercoiling assays

The supercoiling assay was performed following a previously published protocol [37].

## Results

### Oligomeric state of the tagless HU PG0121 protein

**Figure 4:**
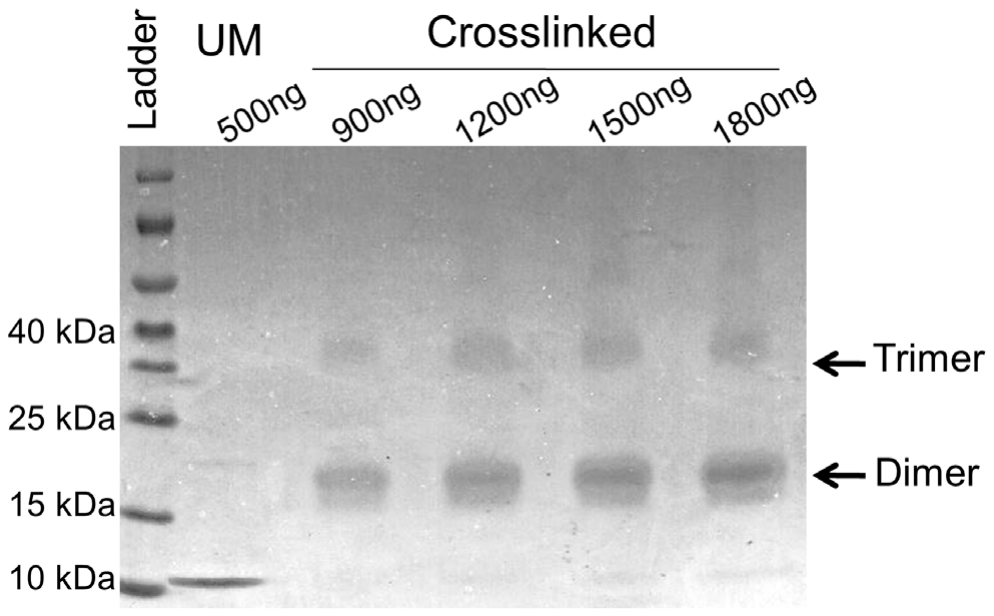
Chemical crosslinking of HU PG0121. HU PG0121 protein exists as dimers in solution. Values indicate ng of HU PG0121 protein in lane. UM is unmodified protein. Lanes containing PG0121 cross-linked with glutaraldehyde are indicated. HU PG0121 has a molecular mass of ∼10 kDa. Arrows indicate the multimeric forms of HU PG10121.

The results of the crosslinking experiment indicated that the HU PG0121 protein exists predominantly as a dimer in solution at all of the tested protein concentrations (Figure 4). Even at the lowest molar protein concentration tested (4.5 µM), the primary species is a dimer. As the protein concentration increases, multimeric species begin to form, as indicated by the accumulation of discrete higher molecular weight bands. Similar to the HU PG0121 protein, HU-like proteins from *Helicobacter pylori* (Hpy-HU) [41] and *Deinococcus radiodurans* (Dr-HU) [37] also exist predominantly as dimers in solution. Because endogenous HU can exist in other species at concentrations up to or exceeding 10 mM depending on the growth conditions [42], HU PG0121 may be present as dimers in the cells.

### Characterization of the tagless HU PG0121 protein and its interaction with DNA

To analyze the binding characteristics of the tagless HU PG0121 protein with the inverted repeat region upstream of the *P. gingivalis* capsule operon, we utilized the 77-bp palindromic sequence in several different DNA configurations that could form at that particular region of DNA (Figure 2). We hypothesized that the HU PG0121 protein might regulate the transcription of the K-antigen capsule operon by binding to the 77-bp stem region of the stem loop structure *in vivo.* The 77-bp inverted repeats are not identical and contain 8 naturally occurring mismatches, including one pair that is separated by 9 bps (Figure 2); this distance corresponds to the distance between kinks that are typically introduced by members of the DNABII family. In this work, we will refer to the sense strands of each of the 77-base repeats annealed to each other as the heteroduplex I substrate. Similarly, when the strands are perfectly complemented, we will refer to these substrates as homoduplex substrates. Both the homoduplex I and heteroduplex I DNA substrates were tested using EMSA because both nucleic acid sequence and mis-paired bases affect the resulting DNA structures, which play an integral role in site-specific recognition by DNA binding proteins [43]. Because the antisense strands of the 77-bp inverted repeat region DNA would form structures similar to heteroduplex I but vary in DNA sequence, we also annealed antisense strands. We refer to this substrate as heteroduplex II, and we refer to the perfectly complemented partner as homoduplex II. A complete list of the DNA substrates tested in this study is presented in Figure 3.

**Figure 5:**
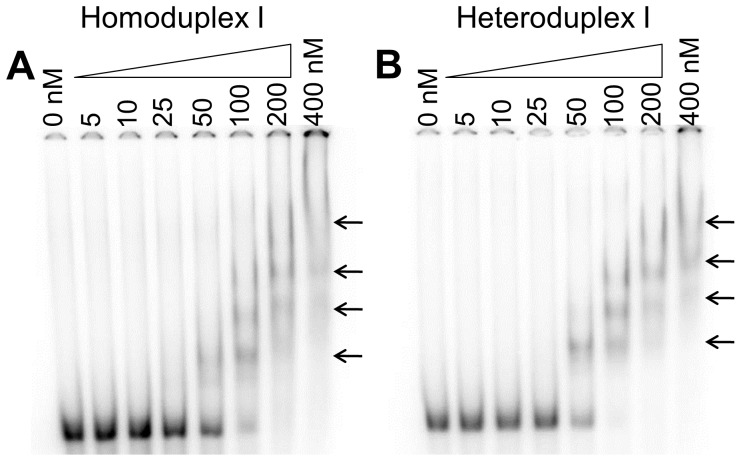
EMSA of HU PG0121 protein and Homoduplex I and Heteroduplex I DNA substrates. 2(Panel A) and Heteroduplex I (Panel B) DNA substrates were incubated with the indicated concentrations of purified HU PG0121 protein, locations of shifted bands are indicated with arrows. The calculated equilibrium dissociation constant (K_d_) values for the binding of HU PG0121 to the Homoduplex and Heteroduplex DNA substrates were 84.0±18.9 nM and 67.5±14.2 nM, respectively.

EMSA testing of the binding of the HU PG0121 protein to the original heteroduplex I DNA probe demonstrated that the introduction of 50 nM protein resulted in the formation of a stable PG0121-DNA complex and that approximately 50% of the free DNA probe was in complex with the protein in the shifted band (Figure 5B). In contrast, although a faint protein-DNA complex was visible upon the addition of at least 50 nM of protein to the homoduplex I DNA (Figure 5A), less than 50% of the free DNA was engaged with the protein to form the shifted product band at this protein concentration. This observation indicates that the HU PG0121 protein had a slightly higher preference for binding to the heteroduplex I DNA probe. The calculated dissociation constants (K_d_) for the HU PG0121 protein binding to the heteroduplex I and homoduplex I DNA probes were 67.5 nM and 84.0 nM, respectively (Figure 3). These results confirmed the binding preference observed in the EMSA experiments. As the concentration of the protein increased, stable higher order protein-DNA complexes were formed, as indicated by the presence of super-shifted bands (Figure 5, 100–400 nM protein concentrations).

To determine the DNA binding characteristics of the HU PG0121 protein, tests were performed using DNA substrates with distorted secondary structures that mimic the preferred substrates for *E. coli* HU and other HU homologs [43], [44] and using single-stranded DNA substrates. These substrates consisted of the 77-bp inverted repeat sequence with variations that were designed to include two 2-nucleotide loops separated by a 9-bp separation, nicks, 1-nucleotide or 2-nucleotide gaps, 3′-overhang, and bulges. Another 77-bp DNA fragment that is unrelated to the 77-bp inverted repeat region was also used as a control. The EMSAs and the resulting K_d_ values demonstrated that HU PG0121 had the highest affinity for binding to DNA substrates that contained loops and bulges, a modest preference for binding to the gap-1 and gap-2 DNA substrates, and the least affinity for the nick and heteroduplex II DNA substrates (Figure 3). Perhaps the most interesting result was that HU PG0121 preferentially binds to the 77-bp inverted repeat region over the non-specific 77-mer DNA substrate. In fact, the non-specific substrate had the highest measured K_d_ value and failed to form a stable nucleoprotein complex via EMSA. In summary, the observed binding preferences of HU PG0121 for DNA substrates with the tested secondary structures can be ranked as followed: Bulges > Loop > Gap-1 > Heteroduplex I > Gap-2 > Homoduplex I > Homoduplex II > 3′-overhang > Heteroduplex II > Nick > 77-mer control.

### Bend permutation assay

**Figure 6:**
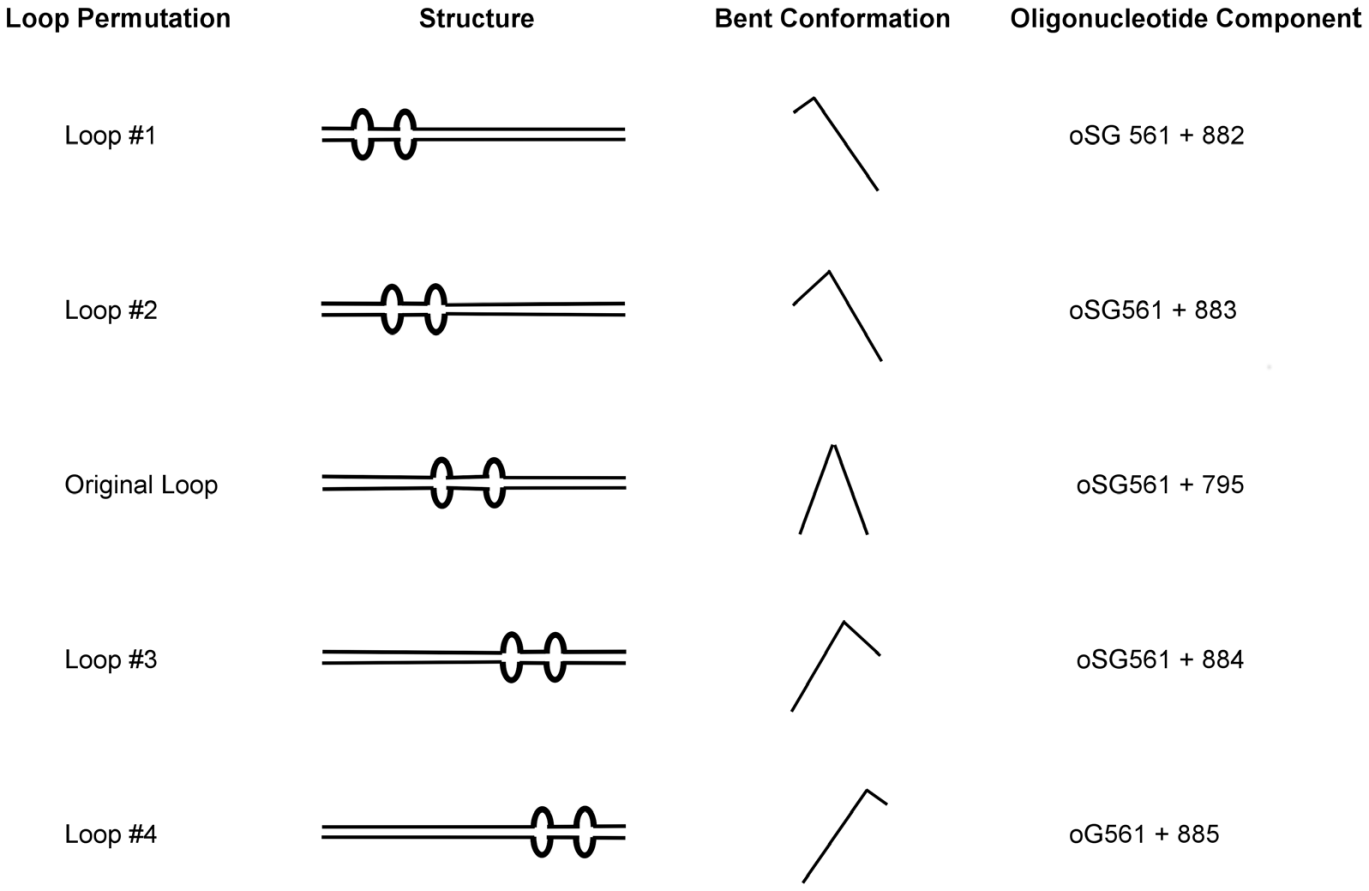
Permutation of the loop structures.

HU protein is considered to be an architectural protein that mediates local DNA distortion because of its ability to bend DNA. To determine whether HU PG0121 can bind bent DNA, and whether the overall DNA probe conformation affects the binding of HU PG0121, EMSAs were performed in which the position of the 2-nucleotide loops were permutated along the 77-bp DNA probe (Figure 6). This results in the formation of DNA probes with different conformations likely because of the variation in the position of the bend. If HU PG0121 has the ability to bind specifically to the loops and bend the DNA, then binding to the probe with centrally located loop structures will result in a DNA conformation that is predicted to migrate the slowest in the gel because it has the smallest mean-squared end-to-end distance [45]. When the loop structures are located closer to the ends of the DNA molecules, the bent DNA conformation that result from protein binding will be more linear (greatest mean-squared end-to-end distance), and this species should migrate faster in the gel.

**Figure 7:**
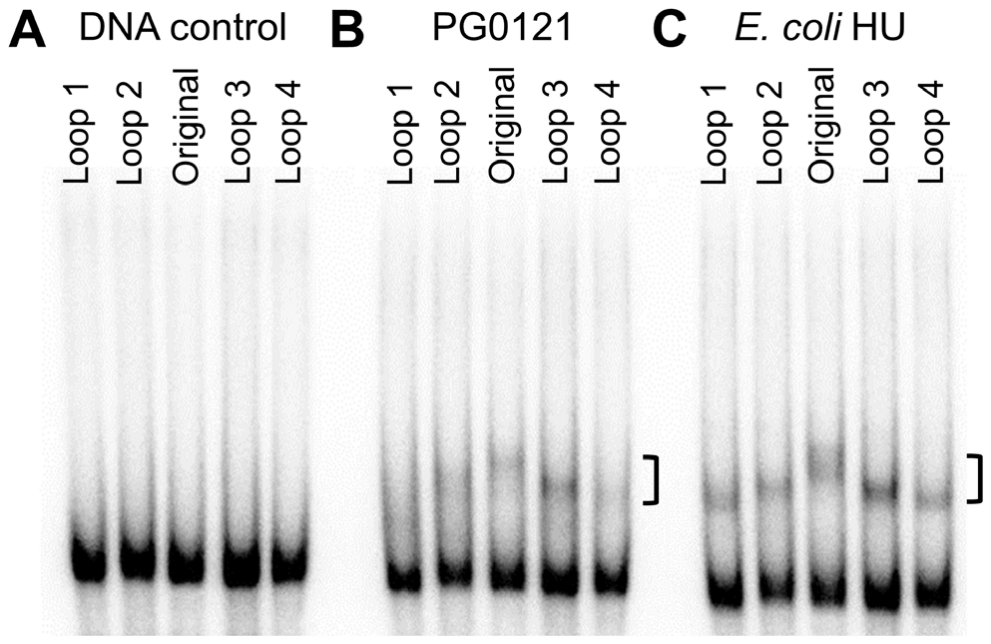
Permutations of the 2-nucleotide loop structure along the inverted repeat sequence. Panel A contains the 2; Panel B, 25 nM HU PG0121; Panel C, 1 nM *E. coli* HU. Lanes are labelled with structures shown in Figure 6. The location of the shifted bands was marked with a bracket.

As shown in Figure 7, HU PG0121 is similar to *E. coli* HU in its ability to bend DNA. The binding of both *E. coli* HU and HU PG0121 to DNA probes with centrally located loop structures resulted in the formation of shifted bands that migrated the slowest in the gel (Figure 7B and C original loop), while the binding to DNA probes with the loops located at the ends of the DNA probe resulted in DNA species that migrated faster. In terms of the affinity of binding, both HU PG0121 and *E. coli* HU proteins showed approximately similar affinity for all the substrates tested. However, HU PG0121 may have slightly lower binding affinity to the probes where the loops are located at the very end of the DNA molecules (Figure 7B loops 1 and 4). Shifted bands are barely visible, and the protein-DNA complexes formed may not be as stable as the rest of the complexes as there is substantial smearing.

### Effects of protein HU PG0121 on the structures and stability of HJ DNA

HJ DNA melting and branch migration assays were done to determine the influence of HU PG0121 on the stability of the overall HJ DNA conformations. The Y DNA substrate used in the branch migration assay is similar to the stem loop structures that may form with transcribed mRNA, another possible substrate for HU PG0121. HJ DNA was analyzed because it is similar to the cruciform structures that may form at the inverted repeat region upstream of the K-antigen capsule operon. The binding of HU PG0121 to either the stem loop or the cruciform structures may be crucial in the regulation of the capsule operon expression.

**Figure 8:**
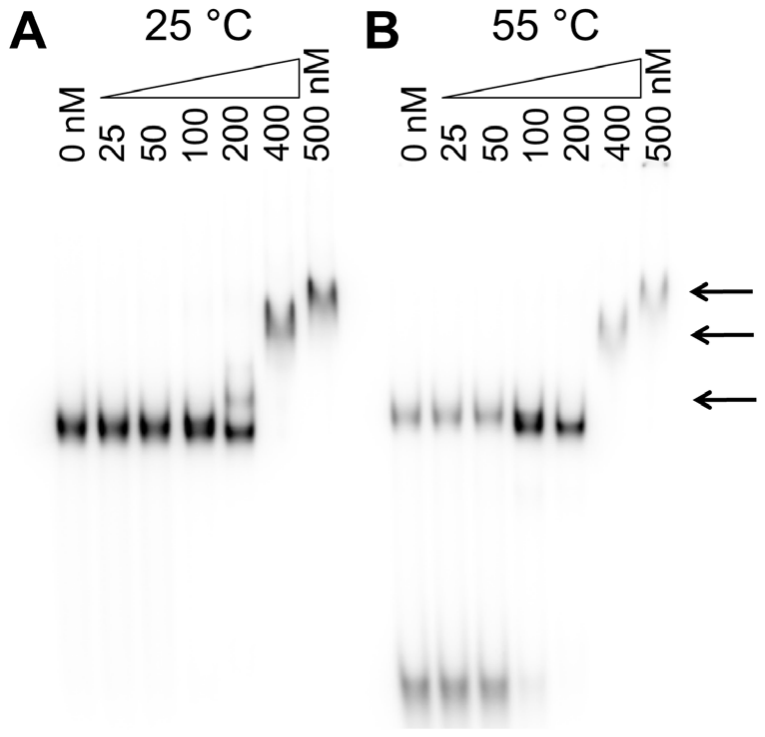
HU PG0121 protects the structure of HJ DNA during thermal denaturation. EMSA was performed in which 25(B) was then heated to 55°C for 10 minutes, while the control group (A) remained at room temperature. Arrows indicate the location of the various DNA-protein complexes.

EMSA showed that one stable DNA-protein complex was formed upon the binding of 200 nM HU PG0121 to the HJ DNA substrate (Figure 8A) with additional complex formation observed at higher protein concentrations. This pattern is similar to that observed for *E. coli* HU binding to HJ DNA [37]. In contrast to *E. coli* HU's significantly higher preference for binding to pre-bent DNA, our data indicate that HU PG0121 does not bind with higher preference to this HJ DNA compared to other DNA substrates tested previously. A significantly higher protein concentration (200 nM) was required for the formation of stable protein-DNA complexes compared to the amount required for other DNA probes (25 nM–100 nM).

Melting assays, which test the ability of a specific protein to protect DNA structures from thermal denaturation, indicate that HU PG0121 can protect and stabilize the HJ structure. As seen from the presence of lower molecular weight species, which coincide with the component oligonucleotides of HJ, (Figure 8B) the absence of HU PG0121 led to the disintegration of HJ DNA structure upon heating. In the presence of HU PG0121, the HJ structure was protected from thermal denaturation with greater protection afforded by higher protein concentrations. Interestingly, there is a loss of the first shifted complex seen at 25°C and 200 nM protein but not at 55°C and 200 nM protein. We suggest that the disappearance of this band is caused by a decrease in the pool of unbound native DNA substrate, shifting the equilibrium away from the bound state. The failure to observe this phenomenon at higher protein concentrations would be the result of a shift in equilibrium in favor of the protein DNA complex(es), an observation consistent with the supershifted complexes observed at higher protein concentrations.

To further explore the interaction of PG0121 at HJ-like structures, a branch migration assay was also performed. DNA branch migration, the isoenergetic exchange of two homologous DNA duplexes through a HJ intermediate, is an important step in genetic recombination [40]. The rate of branch migration has been shown to depend on temperature, ionic conditions, and the type of metal ions present in the reaction[40] because these factors affect HJ folding and structure [46].

**Figure 9:**
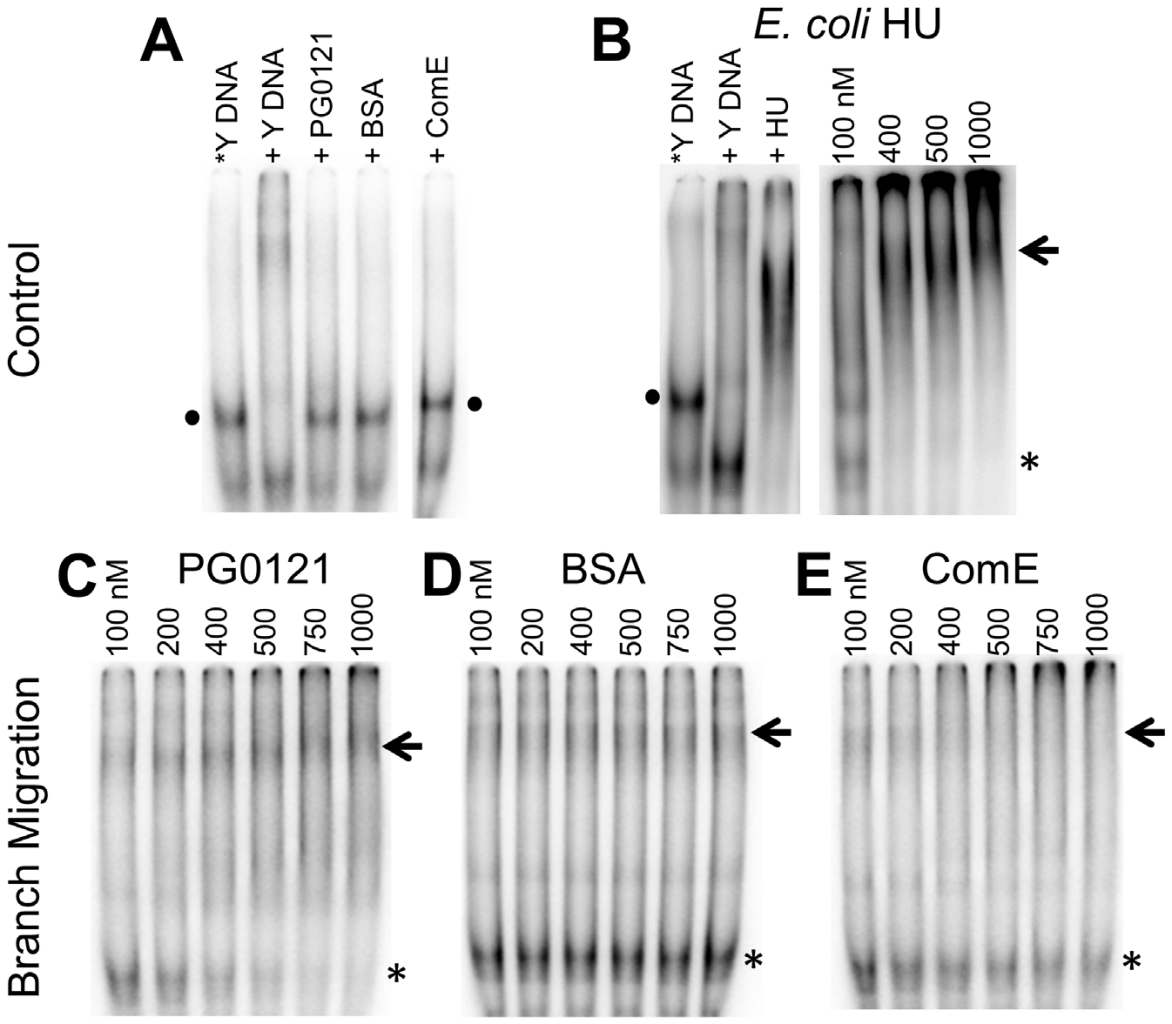
HU PG0121 protein stabilizes the HJ structure. Labeled *Y-DNA controls are shown in Panel A with *Y-DNA alone *Y-DNA mixed with unlabeled Y-DNA, 100 nM PG0121, BSA and ComE. Panel B shows *Y-DNA controls with *E* coli HU protein and a positive control branch migration assay with 100 to 1000 nM *E. coli* HU protein. Branch migration assays with increasing protein concentrations from 100 to 1000 nM for PG0121 (Panel C), BSA (Panel D) and *S. mutans* ComE (Panel E). Arrows indicate the branch migration intermediates, the asterisks indicate the location of the branch migration product bands, and the filled circles indicate the location of the substrate bands.

In a branch migration assay, there are 3 potential outcomes; that HU PG0121 interferes with the formation of the HJ, that it increases the rate of branch migration, or that it impedes the rate of branch migration. If the addition of HU PG0121 protein led to the stabilization of the HJ structure, the HJ structure should become more rigid and progress of branch migration would be blocked. Figure 9 shows the results of this assay, in which HU PG0121 was compared to BSA and *Streptococcus mutans* ComE (a heterologous transcription factor) protein controls. HU PG0121 protein seemed to stabilize the HJ structure and impede branch migration because while branch migration intermediates can clearly be seen, product bands greatly diminished (Figure 9C. On the other hand, the formation of product bands indicates that increasing concentrations of BSA (Figure 9D) or ComE (Figure 9E) does not hinder the movement of the HJ during branch migration.

### Supercoiling of plasmid DNA

**Figure 10:**
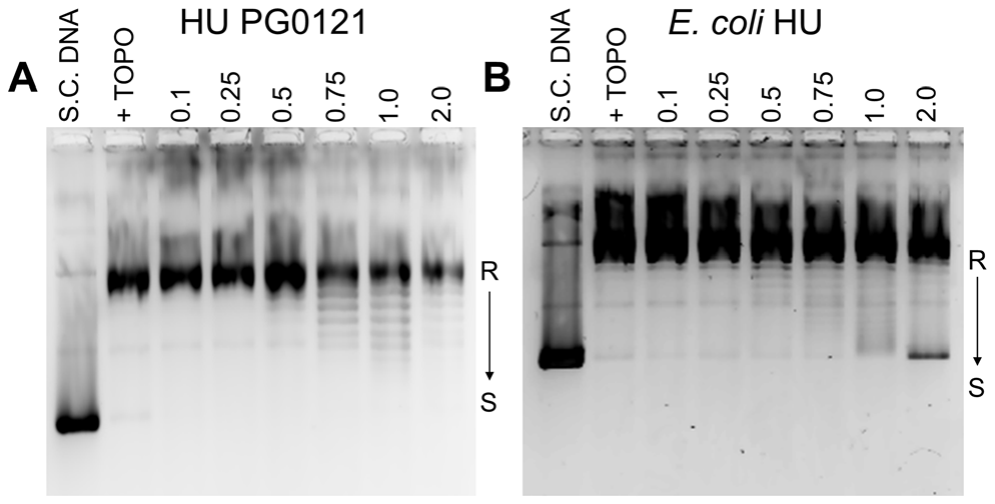
Supercoiling of plasmid DNA by HU PG0121 and *E.* coli HU. Lanes from left: supercoiled plasmid pTrcHis2B DNA (S.C. DNA), relaxed plasmid DNA with topoisomerase I (+ TOPO), addition of increasing micromolar amounts of HU PG0121 (Panel A) or *E. coli* HU (Panel B). Relaxed plasmid DNA is indicated by an R, arrow indicates an increasing degree of DNA supercoiling (S) as protein concentration was increased.

*E. coli* HU shares the ability with eukaryotic histones to introduce negative supercoiling into relaxed circular DNA in the presence of topoisomerase I *in vitro* [47]. Other HU homologs also share this ability with a wide range of efficiencies depending upon the species. The ability of HU PG0121 protein to initiate supercoiling in relaxed circular DNA was tested in supercoiling assays. Compared to *E. coli* HU (Figure 10B), HU PG0121 protein does have a modest ability to introduce supercoils into plasmid DNA relaxed with topoisomerase I (Figure 10A). The introduction of *E. coli* HU to the supercoiling assay reactions results in the formation of a higher superhelical density. Interestingly, the addition of 2 µM of HU PG0121, the highest concentration tested, decreased the amount of supercoiling introduced to the relaxed DNA (Figure 10A, Lane 8); similar results have previously been observed and have been attributed to the inhibition of supercoiling by excessive amounts of HU protein [10].

## Discussion

HU PG0121 shares a 76.7% sequence similarity to the *E. coli* HU β-subunit [48]. HU PG0121 also has the ability to complement some functions of HU in *E. coli* HU double mutants [35]. This high sequence similarity and some functional homology lead to the expectation that HU PG0121 has similar characteristics to other HU proteins in terms of its DNA substrates preference, binding characteristics, and its architectural roles *in vivo*. There are indeed some shared characteristics (such as readily formed dimers), but there are also many distinct characteristics that differentiate HU PG0121 from other HU-like proteins. HU PG0121 may be another example of the divergence in the DNABII family of DNA binding proteins.

### The non-discriminatory binding of HU PG0121 to various DNA substrates argue against its role in DNA repair mechanisms *in vivo*

In terms of its DNA binding preference, the tagless version of *P. gingivalis* HU PG0121 protein demonstrates no significant DNA binding preference to DNA structures with internal flexure points, such as the mismatches, nicks, overhangs, and the HJ DNA, all of which may form at the inverted repeat region upstream of the capsule operon (See Figure 2 for a schematic of these structures). HU PG0121 binds with approximately similar affinity to all of the DNA probes tested, except for a modest preference to the loop and bulges DNA probes (Figure 3). These unusual binding characteristics of HU PG0121 are indeed a striking contrast to *E. coli* HU, which binds to these distorted DNA structures with a dissociation constant that ranges between 1–10 nM at stringent high salt conditions [15], [16] as opposed to the micromolar binding affinity to linear ds-DNA [3]. These observations are not consistent with HU PG0121 binding to DNA repair intermediates *in vivo* like *E. coli* HU, which has been shown to bind to DNA repair and recombination intermediates [16]. The effects of heterodimeric HU-αβ still needs to be determined and could function differently from the homodimeric HU PG0121 protein.

This family of proteins can affect DNA structure upon binding. From the structural data of other family members, HU protein forms a compact core structure of intertwined monomers from which two anti-parallel β ribbons extend to engage a DNA helix [49], [50]. Highly conserved proline residues (Pro63 in PG0121) at the tips of the β-strands mediate two sharp DNA kinks. Presumably, the more the DNA approximates the final bent structure, the stronger the protein binding will be because less energy will be required for creating the contorted bent configuration. The fact that HU PG0121 does not exhibit a significant preference to DNA substrates with intrinsic flexure points or pre-bent configurations compared to the perfect homoduplex substrate suggests that the binding of HU PG0121 to these non-native DNA molecules affords no additional contribution to DNA binding affinity. However, HU PG0121 may exhibit some degree of sequence specificity because of its higher binding affinity to the 77-mer derived from the inverted repeat region compared to the control 77-mer that is not associated with the K-antigen capsule operon.

The size of the DNA constructs used may also account for the similar binding patterns of HU PG0121. It is possible that the protein binds to other regions on the DNA construct to cause the formation of the shifted bands during EMSA. Calculated positive cooperativity values for HU PG0121 binding to all but one of the DNA substrates tested (Figure 3), albeit modest, may also contribute to the uniform binding pattern seen in EMSA (Figure 3). Moreover, it is also possible that HU PG0121 binds and introduces kinks in the DNA that are not 9 bps apart like other DNABII family members. Synthesizing DNA substrates with discontinuities in the structures that are separated by various distances may provide insight into the binding mode of HU PG0121.

### HU PG0121's ability to introduce supercoils into relaxed DNA, may contribute to the regulation of the expression of capsule operon

HU PG0121 has the ability to introduce supercoiling into relaxed plasmid DNA similar to *E. coli* HU, but at a lower efficiency. This suggests a possible role of HU PG0121 in *P. gingivalis* bacterial genome compaction *in vivo*. Addition of excess HU protein causes a partial inhibition of supercoiling, while an equimolar ratio of DNA to HU has been shown to compact DNA and protect it from relaxation *in vitro* [11]. In *E. coli*, the homodimeric HU-ββ is not capable of promoting DNA supercoiling, while the homodimeric HU-αα and the heterodimeric HU-αβ are [4]. The abilities of *P. gingivalis* HU-αα (PG1258, by annotation [28]) or *P. gingivalis* HU-αβ to induce supercoiling in DNA have not yet been determined. However, the fact that *P. gingivalis* HU-ββ is able to introduce supercoiling into relaxed DNA may serve as another example of the divergent characteristics of HU PG0121.

The capacity to restrain supercoils by HU PG0121 may inhibit the formation of cruciform structures at the inverted repeat region upstream of the K-antigen capsule region because this particular DNA conformation is preferably formed in the presence of negatively supercoiled DNA [26]. Even though the cruciform formation is not energetically favorable [51], several studies have shown that a significant proportions of known palindromes exist as cruciform extrusions *in vivo*, particularly due to negative supercoiling [52]–[54]. This includes the effects of traversing transcriptional complexes to leave high densities of negative supercoils in their wake [55]. HU PG0121 could absorb these negative supercoils preventing cruciform extrusion.

Alternatively, HU PG0121 could act to stabilize or even induce the formation of the cruciform. PG0104, the gene immediately upstream of the capsule operon has a 57% amino acid similarity to *B. subtilis* topoisomerase III [28]. This protein, together with HU PG0121, could facilitate the formation of DNA supercoiling. *In vitro*, HU PG0121 has been demonstrated to stabilize HJ DNA structures, impede the progression of branch migration, and protect HJ DNA from thermal denaturation. This interaction with cruciform structures is significant because it may influence the regulation of expression of the K-antigen capsule operon in *P. gingivalis* in a similar manner to how *E. coli* HU is implicated in the autoregulation of *hupA* genes [26]. *E. coli* HU increases the negative superhelical density at the promoter region to facilitate the formation of the cruciform structures [26] which may block the replication machinery access and/or transcription initiation signals, thus repressing the expression of the *hupA* gene. The initiation of transcription of the *E. coli hupA* gene is negatively regulated by the conformational change of the functional promoter domains due to the formation of the cruciform structure [26]. Cruciform structure formation upstream of the capsule operon start codon in *P. gingivalis* may cause changes to the overall DNA conformation at the promoter region in a similar manner. These changes may subsequently block access of the transcription machinery to the promoter initiation signals. Therefore, PG0121 may contribute to the negative regulation of the expression of the capsule operon. Given that the absence of HU PG0121 reduces gene expression, we favour the first model of cruciform suppression.

In addition to facilitating the formation of cruciform structures, supercoiling of DNA has been shown to influence many other DNA processes, such as DNA replication, recombination, and condensation by assisting in the formation of higher order DNA-multiprotein complexes [56]–[60]. These higher order DNA-protein complexes have in turn been shown to be involved in the repression of gene expression. The repression of *E. coli gal* promoters, for example, involves the formation of repression complex consisting of Gal repressor (GalR), HU, and supercoiled DNA [61]. Supercoiling has been argued to help in the formation of the transcription repression complex by influencing the overall 3-dimensional geometry of the DNA, allowing for correct alignment of the proteins with the appropriate DNA sites [61]. Likewise, supercoiling of DNA induced by HU PG0121 may also initiate the formation of higher order DNA-multiprotein complexes that may help in the regulation of the expression of the capsule operon in *P. gingivalis*. In this case, HU PG0121 may also coordinate the communication between other as yet unknown proteins that are involved in the complex formation.

If indeed the supercoiling of DNA induced by HU PG0121 can manipulate the 3-dimensional geometry of the DNA, it would suggest that HU PG0121 plays an architectural role *in vivo*. The architectural roles of HU are one of the characteristics shared by almost all of the HU homologs studied to date and include bending the DNA and inducing specific DNA topology to promote assembly of higher order nucleo-protein structures [36]. The result of the permutation assay, which was performed to test the architectural ability of HU PG0121 in bending DNA, shows that HU PG0121 does have the ability to bend DNA with little preference for repair intermediates. It is possible that HU PG0121 only bends DNA by virtue of the final architecture of the DNA substrates; we think this unlikely given the ability of PG0121 to restrain supercoils where bending is a necessary requirement.

### The expression of the capsule operon may be regulated through the binding of HU PG0121 to RNA structures

Another mechanism through which HU PG0121 could regulate the expression of the K-antigen capsule operon is through an interaction with RNA. Although the ability of HU PG0121 to bind to RNA has yet to be tested, other HU homologs have been shown to have the ability to bind RNA molecules. Because of this ability, HU may have additional regulatory functions within the cell [22], [23]. Furthermore, the binding of HU PG0121 to the related DNA architectures has been tested and showed no obvious preference.

In *E. coli*, HU has been demonstrated to bind to the upstream region of *rpoS* mRNA, encoding the RNA polymerase stress sigma factor, by virtue of the secondary structures of the RNA. This binding stimulates the translation of the mRNA, which consequently results in an increased expression of the RpoS protein [22]. The expression of the K-antigen capsule may also be regulated through a similar mechanism. There is a high predicted probability that the inverted repeat region forms a stem loop structure in the mRNA [28]. The complex secondary structures of the RNA may serve as the recognition motifs for the binding of HU to RNA [23]. The binding of HU PG0121 to these RNA secondary structures may then alter the RNA sufficiently to allow transcriptional read through, and the subsequent translation, through a mechanism known as anti-termination [28]. Recently, the importance of this regulatory mechanism in the regulation of the expression of large polysaccharide synthesis operons has been validated by a study on a close relative of *P. gingivalis*; *Bacteroides fragilis* [62]. The binding of HU PG0121 may also contribute to the increase in the expression of the K-antigen capsule by enhancing the stability of the RNA transcripts [28].

### Exclusivity of PG0121: Possible Role of PG1258 in Capsule Expression

Although HU-β homodimers seemed to be sufficient to perform some of the DNA related functions tested in this study, it is possible that *P. gingivalis* HU may be present as a heterodimer *in vivo*. In *E. coli*, the three forms of HU do not behave identically in terms of their functions *in vivo*. For example, the homodimeric HU-ββ form of HU is not capable of promoting DNA supercoiling, while the homodimeric HU-αα and the heterodimeric HU-αβ are [4]. The heterodimeric form of HU has also been shown to be required for optimal *E. coli* survival after prolonged starvation [4]. An *E. coli hupA* mutant exhibits a slightly higher sensitivity compared to the *hupB* mutant, which in turn is more sensitive than *E. coli* WT strain upon exposure to ionizing radiation [5].

*P. gingivalis* has been shown to also encode an *E. coli hupA* homolog, PG1258, which shares a 71.1% amino acid similarity to *E. coli* HupA [48]. Previous studies have suggested that PG1258 is essential because no PG1258 mutant can be generated [28]. It is possible that the expression of PG1258 (HU-α) may be required for the proper functioning of HU PG0121 (HU-β) in its role as one of the regulators of the capsular gene expression. Although it is also possible that PG0121 functions as independent homodimers in capsule synthesis without the involvement of PG1258, further analysis is needed to determine the possible involvement of PG1258 in capsular gene expression.

In summary, with its unique DNA binding properties, HU PG0121 may act as an accessory factor that affects the transcription of the K-antigen capsule operon. Other factors that have yet to be identified may need to be present and interact with HU PG0121 to ensure an efficient binding and functioning of this HU-like protein. More studies are needed to determine the exact mechanisms utilized by HU PG0121 in the regulation of K-antigen capsule expression. A greater understanding of how *P. gingivalis* controls and coordinates expression of surface polysaccharides will offer a deeper understanding of the roles this organism plays in the formation of periodontitis, and will hopefully be beneficial in reducing the occurrence and severity of periodontitis cases.
